# O-6-Methylguanine-DNA methyltransferase expression is associated with pituitary adenoma tumor recurrence: a systematic meta-analysis

**DOI:** 10.18632/oncotarget.14936

**Published:** 2017-02-01

**Authors:** Congxin Dai, Bowen Sun, Xiaohai Liu, Xinjie Bao, Ming Feng, Yong Yao, Junji Wei, Kan Deng, Chengxian Yang, Xueyuan Li, Wenbin Ma, Renzhi Wang

**Affiliations:** ^1^ Department of Neurosurgery, Peking Union Medical College Hospital, Chinese Academy of Medical Sciences & Peking Union Medical College, Beijing 100730, China

**Keywords:** pituitary adenomas, MGMT, recurrence, aggressive

## Abstract

O-6-methylguanine-DNA methyltransferase (MGMT) reportedly counteracts the cytotoxic effects of the alkylating agent temozolomide. MGMT expression is often low in aggressive pituitary adenomas (PAs) and recurrent PAs. However, because these associations are controversial, we performed this meta-analysis to clarify the involvement of MGMT in the prognosis and clinicopathology of PA. We searched for relevant studies in electronic databases (MEDLINE, the Cochrane Library Database, EMBASE, CINAHL, Web of Science and the Chinese Biomedical Database (CBD)) and calculated/pooled the odds ratios (ORs) or standard mean differences (SMDs) with 95% confidence intervals (95% CIs). Eleven case-control studies with a total of 454 PA patients were included. Our meta-analysis revealed that lower expression of MGMT was associated with PA recurrence (OR=2.09, 95% CI=1.09–4.02; *p*=0.026). On the other hand, MGMT expression was not associated with PA invasiveness (OR=1.112, 95% CI=0.706–1.753; *p*=0.646), Unexpectedly, MGMT expression could not be used to distinguish functional from non-functional PA patients (OR=1.766, 95% CI=0.938–3.324; *p*=0.078). The MGMT expression was not found to be related to other clinicopathological indicators of PA including age, gender or tumor size. No publication bias was detected in this meta-analysis (p>0.05). This meta-analysis suggests that MGMT expression may be associated with PA tumor recurrence, but not be related to invasiveness or other clinicopathological indicators. Thus, detection of MGMT expression may facilitate outcome prediction and guide clinical therapy for PA patients.

## INTRODUCTION

Pituitary adenomas (PAs) are the second most common type of intracranial neoplasm and account for approximately 15% of intracranial tumors, although autopsy studies indicate a higher incidence of 25% [1, 2]. Most PAs are benign and exhibit no expansive properties; however, approximately 30-40% are aggressive PAs that massively invade the surrounding anatomical structures [3]. Surgery has long been the first-line treatment for PAs (except for prolactinomas). However, aggressive PAs are difficult to resect completely and tend to recur due to massive invasion of adjacent tissues (e.g., the cavernous sinus and the dura). Such PAs are generally refractory to repeated surgeries, radiotherapy and alternative medical therapies [4]. Therefore, these aggressive PAs are difficult to manage and are associated with poor prognosis and fatality [5].

Temozolomide (TMZ), a routine chemotherapy for glioblastoma (GBM), it exerts its cytotoxic activity by alkylating DNA at the O6 position of guanine. In recent decades, TMZ has been reported to have significant therapeutical effects on PAs and pituitary carcinomas [6, 7]. However, O-6-methylguanine DNA methyltransferase (MGMT), a DNA repair protein, alters the methylation status of DNA and reverses TMZ-induced alkylation. Until now, MGMT has been recommended as an important predictor of the efficacy of TMZ therapy in GBM [8, 9]. Several clinical studies have demonstrated that the expression and/or promoter methylation of MGMT may have prognostic significance in GBM [10–12]. The low expression of MGMT has also been reported with a higher frequency amongst PAs [13]. Some studies have indicated that MGMT expression is associated with PA patients’ prognoses and responses to TMZ [14–16]. However, in other studies, MGMT expression has not correlated significantly with clinical responses to TMZ or clinical outcomes in patients with PAs [17–20]. It remains uncertain whether discrepancies in these data are mainly due to limited sample sizes or genuine heterogeneity.

Thus, it is necessary to review and systematically assess the precise association of MGMT expression with the prognoses and clinicopathological indicators of PAs patients. To this end, we have performed the following meta-analysis.

## RESULTS

### Search results and characteristics of included studies

**Figure 1:**
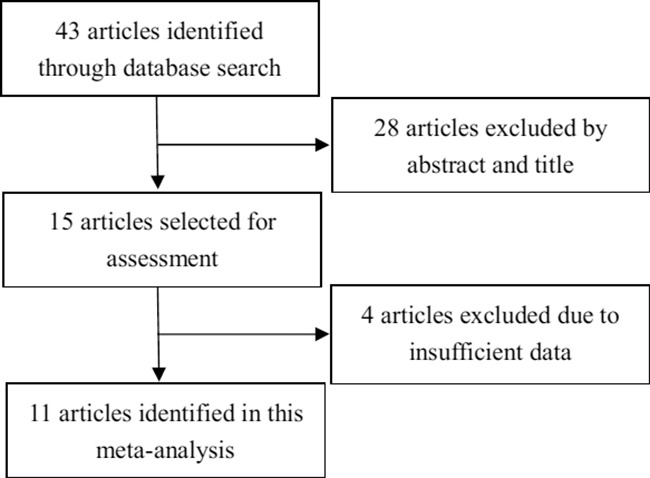
Flow chart depicting the study selection procedure Eleven studies were included in this meta-analysis according to the inclusion criteria.

As shown in Figure 1, 43 papers were initially identified in our search. During the initial review of the titles and abstracts, 28 articles not relevant to our goal were excluded. Two reviewers then independently reviewed the remaining 15 articles, 4 of which were excluded for insufficient data. Ultimately, 11 articles that met the criteria were included.

The general features of the 11 articles are summarized in Table 1. A total of 454 patients with PA were involved in this meta-analysis, including 180 patients with invasive PAs and 243 patients with noninvasive PAs (the invasion properties of PA were not mentioned for the remaining patients). The classical immunohistochemistry (IHC) method was performed to detect MGMT protein expression in all included studies. The percentage of positive MGMT expression ranged from 5 to 90%. Positive MGMT-expressing patients were defined in three ways. Most investigators scored MGMT expression as positive if any part of the nucleus or cytoplasm was stained. In these studies, MGMT immunoexpression was scored on a 4-tiered scale (1=negative or limited to 10%, 2=10–25%, 3=25–50% and 4=≥50%). Scores of 1 and 2 were combined to form the category of “low level MGMT expression,” while scores of 3 and 4 represented intermediate and high MGMT expression, respectively [26, 27, 31]. There were also differences in the definition of the cut-off value of high MGMT expression; some investigators defined the cut-off value using a score combining the intensity and percentage of MGMT expression, while others used only the percentage of stained cells.

**Table 1:** Baseline characteristics of studies included in the meta-analysis

Study ID	Country	Year	Num.	Gender (M/F)	Age (years)	Invasiveness Yes/No	Recurrence Yes/No	Method	Cut-off
Takeshita A [21]	Japan	2009	24	3/21	–	9/15	–	IHC	5%
McCormack A I [22]	Australia	2009	88	–	–	46/42	13/75	IHC	10%
Widhalm G [23]	Austria	2009	45	29/16	–	25/20	24/21	IHC	50%
Salehi F [24]	Canada	2010	8	3/5	62.4(57–66)	–	–	IHC	25%
Fealey M E [25]	USA	2010	23	15/8	35.0(17–69)	–	2/21	IHC	25%
Lau Q [26]	USA	2010	30	–	–	15/15	–	IHC	Score>3
Zuhur S S [27]	Turkey	2011	25	10/15	43.0(23–65)	10/15	–	IHC	Score>3
Salehi F [28]	USA	2011	12	8/4	34.4(17–64)	7/5	2/9	IHC	10%
Salehi F [29]	USA	2012	40	12/28	40.6(15–62)	16/24	11/29	IHC	25%
McCormack A [30]	Australia	2013	21	15/6	55.4(24–79)	9/12	2/19	IHC	10%
Jiang X [31]	China	2013	138	67/71	44±14.5	43/95	12/126	IHC	Score≥3

Num: numbers; M: male; F: female; IHC: immunohistochemistry;

### Study assessment

The quality of each eligible study, as assessed with the European Lung Cancer Working Party criteria, is presented in Table 2. The mean final score of the all studies was 67.25%, and the global scores of studies analyzing recurrence and invasiveness were 67.7% and 69.5%, respectively.

**Table 2:** Clinical and methodological characteristic of included studies

	No.of studies	Design	Method	Generalizability	Results analysis	Global score(%)
All studies	10	6.7	7.1	6.9	6.2	67.25
Invasiveness	7	7.2	6.8	6.5	7.3	69.5
Recurrence	7	6.6	6.9	6.5	7.1	67.75

### Quantitative data synthesis

**Figure 2:**
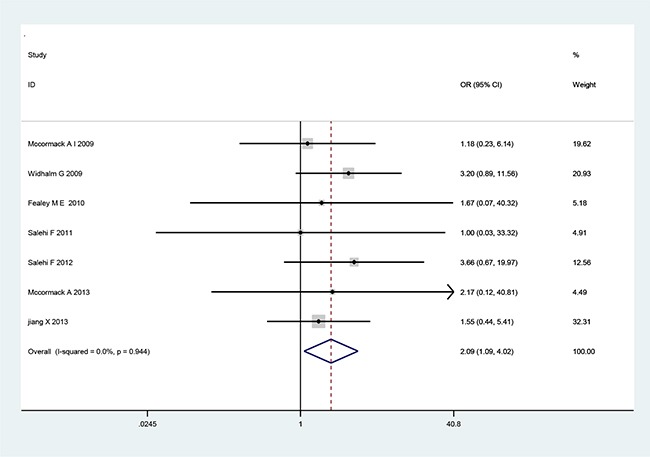
Forest plots for the relationship between MGMT expression and PA tumor recurrence The pooled OR for all seven studies was 2.09 (95% CI 1.09–4.02; *p*=0.026). No heterogeneity was observed (*χ^2^*=1.71, *p*=0.944, *I*^2^=0).

**Figure 3:**
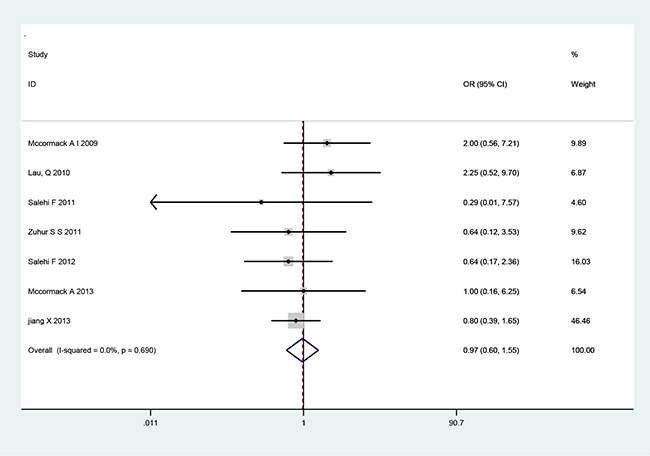
Forest plots for the relationship between MGMT expression and PA tumor invasiveness The pooled OR for all seven studies was 1.11 (95% CI 0.71–1.75; *p*=0.646). No heterogeneity was observed (*χ^2^*=8.5, *p*=0.279, *I*^2^=19.1).

There are seven cohort studies that referred to the relationship of MGMT expression with tumor recurrence or invasiveness pituitary adenoma (Table 3). A fixed-effects model was used because there was not significant heterogeneity among the studies (Table 3). The pooled OR from all seven studies on recurrence was 2.09 (95% CI: 1.09–4.02; *p*=0.026) (Figure 2), indicating that low MGMT expression predicted recurrence and poor survival in PA patients. No heterogeneity was observed (*χ^2^*=1.71, *p*=0.944, *I*^2^ =0) (Table 3). However, we found no significant association between MGMT expression and PA invasiveness from all seven included studies regarding to invasion (OR=1.11, 95% CI=0.71–1.75; *p*=0.646) (Figure 3), indicating that MGMT may be not involved in the invasion of PA. There was no significant heterogeneity among the studies (*χ^2^*=8.65, *p*=0.279, *I*^2^ =19.1%) (Table 3). The above results indicate that low expression of MGMT may be related to tumor recurrence but not invasion of patients with PAs.

**Table 3:** Pooled HR and 95% CI in meta–analysis of association of MGMT expression with chinicopathological indicators

	No.of results	OR	95% CI	heterogeneity		
				*χ^2^*	*p*	*I^2^* (%)
Age	7	0.99	0.57–1.74	6.78	0.342	11.5
Gender	7	1.08	0.63–1.85	5.05	0.538	0
Invasiveness	7	1.11	0.71–1.75	8.65	0.279	19.1
Tumor size	4	0.94	0.50–1.78	9.32	0.025	67.8
Recurrence	7	2.09	1.09–4.02	1.71	0.944	0
Functional	3	1.97	0.94–3.32	2.05	0.360	2.2

**Figure 4:**
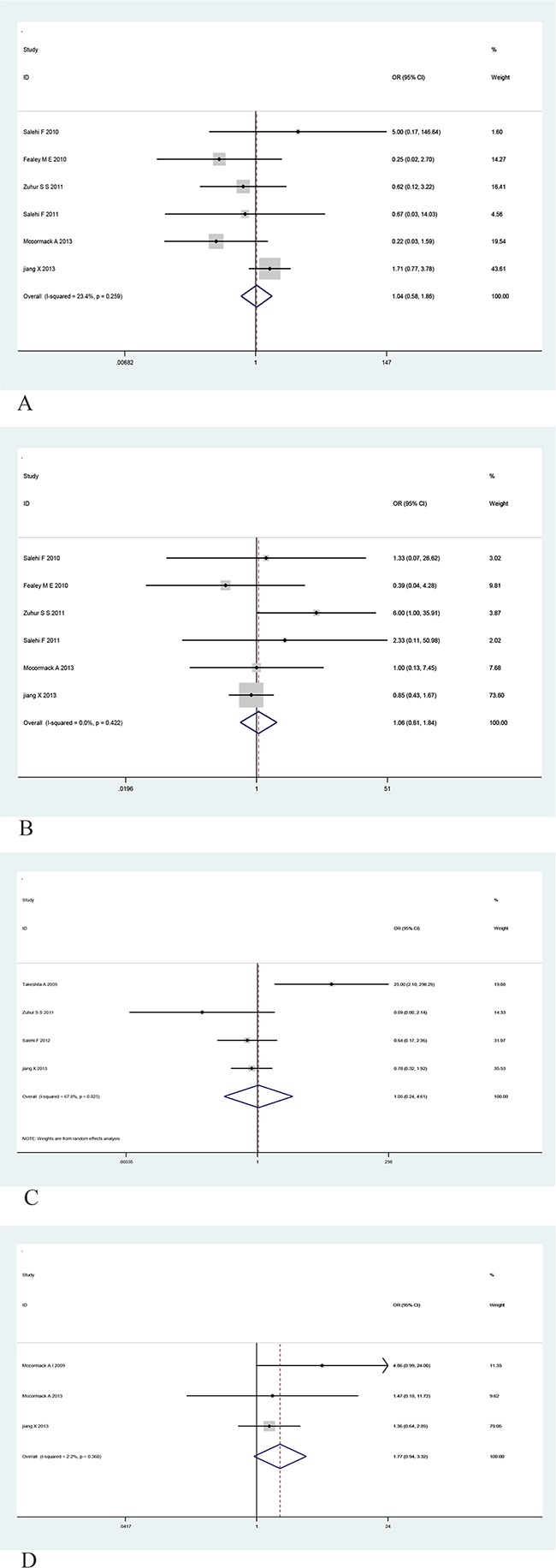
Forest plots for the relationship between MGMT expression and age, gender, tumor size and functional status of PA

To gain further insight into the value of MGMT as a biomarker, we investigated the association of MGMT expression with various clinicopathological indicators, including age (greater than the median age), gender, tumor size, and functional status (Table 3). However, no significant relationship was observed between MGMT expression and age, gender, tumor size, or functional status (Figure 4A, 4B, 4C and 4D). To summary, these findings indicate that lower MGMT expression may be used to predict the recurrence of PAs, however, MGMT expression is not related to other clinicopathological indicators, such as invasiveness age, gender, tumor size, or functional status of patients with PAs.

### Publication bias

**Figure 5:**
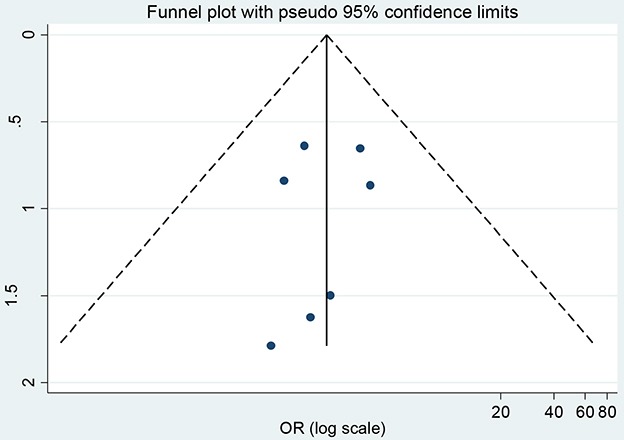
Begger's funnel plots of the association between MGMT expression and PA tumor recurrence

**Figure 6:**
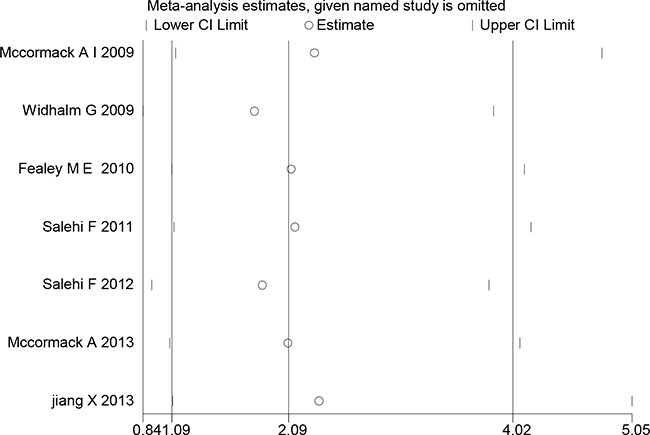
Sensitivity analysis of the association between MGMT expression and PA tumor recurrence Results were computed through the omission of each study in turn. Meta-analysis fixed-effect estimates were used. The two ends of the dotted lines represent the 95% CI.

In the present meta-analysis, the publication bias among studies with regard to tumor recurrence was investigated with Begg's and Egger's tests. No publication bias was observed (*p*=0.124, 0.610, respectively). The shapes of Begger's and funnel plots did not reveal any obvious asymmetry (Figure 5). Sensitivity analysis was performed to assess the effects of individual studies on the pooled ORs through omission of each individual study. No individual study significantly affected the pooled ORs (Figure 6), indicating that the results were statistically robust.

## DISCUSSION

Aggressive PAs are notoriously difficult to manage and are associated with poor prognosis because the therapeutic options are limited and the tumors are generally refractory to standard therapy [32]. Despite the use of multimodal therapies, including repeated surgeries, radiotherapy and alternative medical therapies, postoperative recurrence often occurs [33, 34]. Therefore, it is essential to identify novel molecular markers that will allow the early prediction of PA recurrence and/or invasiveness and the early application of multi-modal therapy to prevent tumor recurrence.

As a salvage therapy, TMZ has recently been shown to have significant efficacy for the treatment of aggressive PAs and pituitary carcinomas [6]. However, some patients acquire TMZ resistance after treatment. MGMT has been suggested as a biomarker that predicts the response to TMZ and the prognosis in GBM [9, 12]. Despite the large number of studies on this subject, the prognostic value of MGMT for predicting PA patient survival is controversial, due to the small sample sizes and genuine heterogeneity of the published studies [23, 27, 30].

In the present meta-analysis, we evaluated the association of MGMT expression with features of human PAs. Ultimately, 11 independent case-control studies with a total of 454 PA patients were included. Our meta-analysis results suggested that lower expression of MGMT was associated with PA recurrence, suggesting that MGMT expression could be used as a marker of poor prognosis and tumor recurrence for patients with PA. Widhalm et al. reported that low MGMT expression was observed more frequently in patients with progressive tumors than in tumor-free subjects [23]. Consistent with this, Salehi et al. also demonstrated that 92% of silent subtype 3 PAs exhibited low MGMT immunoreactivity [28]. Although the exact function of MGMT in the pathogenesis and progression of PAs is not yet fully understood, it may be that low MGMT expression causes the upregulation of gene sets involved in DNA repair and transcription, thus increasing mutagenesis, which further drives the tumorigenic process and increases cellular proliferation [37]. Until now, no study has demonstrated that any molecular marker could be used routinely to predict PA recurrence. Here, we provided the first meta-analysis of the relationship between MGMT expression and biological characteristics of PAs, and found that low expression of MGMT may be associated with PA recurrence, suggesting that TMZ therapy may have promising efficacy for recurrent PAs with low MGMT expression.

Nevertheless, our meta-analysis also had some limitations. MGMT expression in the included studies was measured by IHC, which depends strongly upon methodological factors. The different sources and concentrations of primary and secondary antibodies may have severely reduced the reliability and applicability of the results regarding MGMT expression. Furthermore, there were large differences in the definitions of cut-off values; after all, there are no criteria available for such cut-offs. In addition, the present research was restricted to articles published in English and Chinese, because articles in other languages such as Japanese were not accessible to the readers. Last but not least, this meta-analysis used a retrospective study design that may have led to subject selection bias and thus may have reduced the reliability of our results.

In conclusion, the present meta-analysis indicates that MGMT expression is indeed associated with PA recurrence, but not with invasiveness, age, gender, tumor size or functional status. Thus, MGMT expression may be used as a molecular marker for the early prediction of PA recurrence and the identification of candidates for TMZ therapy. However, due to the limitations mentioned above, further large-scale studies are still required to confirm our findings.

## MATERIALS AND METHODS

### Search strategy

A literature search was carried out with the databases of CISCOM, CINAHL, Web of Science, PubMed, Google Scholar, EBSCO, Cochrane Library, and CBD up to July 2016. There was no language restriction. The following keywords and MeSH terms were used: “pituitary”, “pituitary adenoma”, “pituitary adenomas”, “pituitary tumor”, “pituitary tumors”, “pituitary macroadenoma’’, “MGMT” or “O^6^methylguanine DNA methyl transferase”, etc. We also performed a manual search to find other potential articles.

### Selection criteria

The following criteria were used for the selection of studies for this meta-analysis: (1) the study was designed as a clinical cohort study or case-control study; (2) the study related to the expression of MGMT in human PAs; (3) all patients had confirmed diagnoses of PA; (4) the study provided sufficient information about the MGMT expression and clinical characteristics of the PAs; and (5) the invasiveness of the PAs was assessed by imaging characteristics or intraoperative observation. If a study did not meet the inclusion criteria, it was excluded. The most recent publication or the publication with the largest sample size was included when the authors published several studies using the same subjects. Any disagreements were resolved through discussions and subsequent consensus.

The following criteria were used for the diagnosis of invasive PAs: (1) PAs of grades III and IV and stages C, D, and E were considered invasive according to Hardy classification; (2) invade to adjacent structures such as the parasellar region, cavernous sinus and hypothalamus could be seen on MRI and CT scans; (3) tumor cell invasion was pathologically confirmed in the sellar bone or adjacent dura mater; and (4) the sellar bone and dura mater were invaded and damaged, and tumors penetrated the sphenoid sinus or invaded the parasellar vascular and nervous crossroads. If a tumor did not meet these criteria, it was considered to be a non-invasive PA.

### Data extraction

Two reviewers collected the following data independently using a purpose-designed form: the name of the first author, language of publication, publication date, country of the population studied, sample size, source of subjects, histology, detection methods, MGMT expression, invasion of PAs, recurrence of PAs, etc. Disagreement between the two reviewers was settled by a third reviewer.

### Quality assessment

We evaluated the methodological quality of the included studies by reading and scoring each publication according to the quality scale for biological prognostic factors established by the European Lung Cancer Working Party [38]. This scale is widely used to assess the scientific design, laboratory methodology, generalizability and result analysis of studies. A total of 10 points could be attained in each category, so the maximum total score was 40 points. All reviewers compared their calculated scores and, if necessary, reached a consensus score during a meeting. The final scores represent the percentage of the maximum achievable score, ranging from 0 to 100 percent. Therefore, higher scores indicate better methodological quality.

### Statistical analysis

Meta-analysis was performed with STATA 12.0 software (Stata Corp, College Station, TX, USA). Crude odds ratios (ORs) or standard mean differences (SMDs) with corresponding 95% confidence intervals (95% CIs) were calculated. OR and 95% CI could be extracted directly from included studies. The Z test was used to estimate the statistical significance of the ORs. Cochran's Q-statistic and I^2^ tests were performed to evaluate potential heterogeneity among the studies. A random-effects model was used if the Q-test yielded a *P* value <0.05 or the I^2^ test yielded a value >50%, indicating significant heterogeneity; otherwise, if heterogeneity was not significant, a fixed-effects model was used. Subgroup and meta-regression analyses also were used to explore potential sources of heterogeneity. A sensitivity analysis was carried out, in which each study was omitted in turn, to evaluate the influence of each study on the overall estimate. Funnel plots and Egger's linear regression test were used to investigate publication bias. All *P*-values were two-sided, and *P*<0.05 was considered statistically significant.
